# Supplementary material for: Cortical Cyclin A controls spindle orientation during asymmetric cell divisions in Drosophila
Source: Nat Commun. 2022 May 17;13:2723. doi: 10.1038/s41467-022-30182-1 (PMC9114397; doi:10.1038/s41467-022-30182-1)
Supplement: Supplementary file 1 — Supplementary Information [file 41467_2022_30182_MOESM1_ESM.pdf]

Supplementary information

Cortical Cyclin A controls spindle orientation during asymmetric cell division in *Drosophila*

Pénélope Darnat[1], Angélique Burg[1], Jérémy Sallé[2], Jérôme Lacoste[1], Sophie Louvet-Vallée[1], Michel Gho[1#*] and Agnès Audibert[1 #*]

[1] Sorbonne Université, CNRS, Laboratoire de Biologie du Développement - Institut de Biologie Paris Seine (LBD-IBPS), Cell cycle and cell determination Team, F-75005 Paris, France.

[2] Institut Jacques Monod, Université Paris Diderot/CNRS, Cellular Spatial Organization Team, F-75005 Paris, France.

Table of content:

Supplementary figures ……………………………………………………… p 2

Supplementary tables ……………………………………………………… p 10

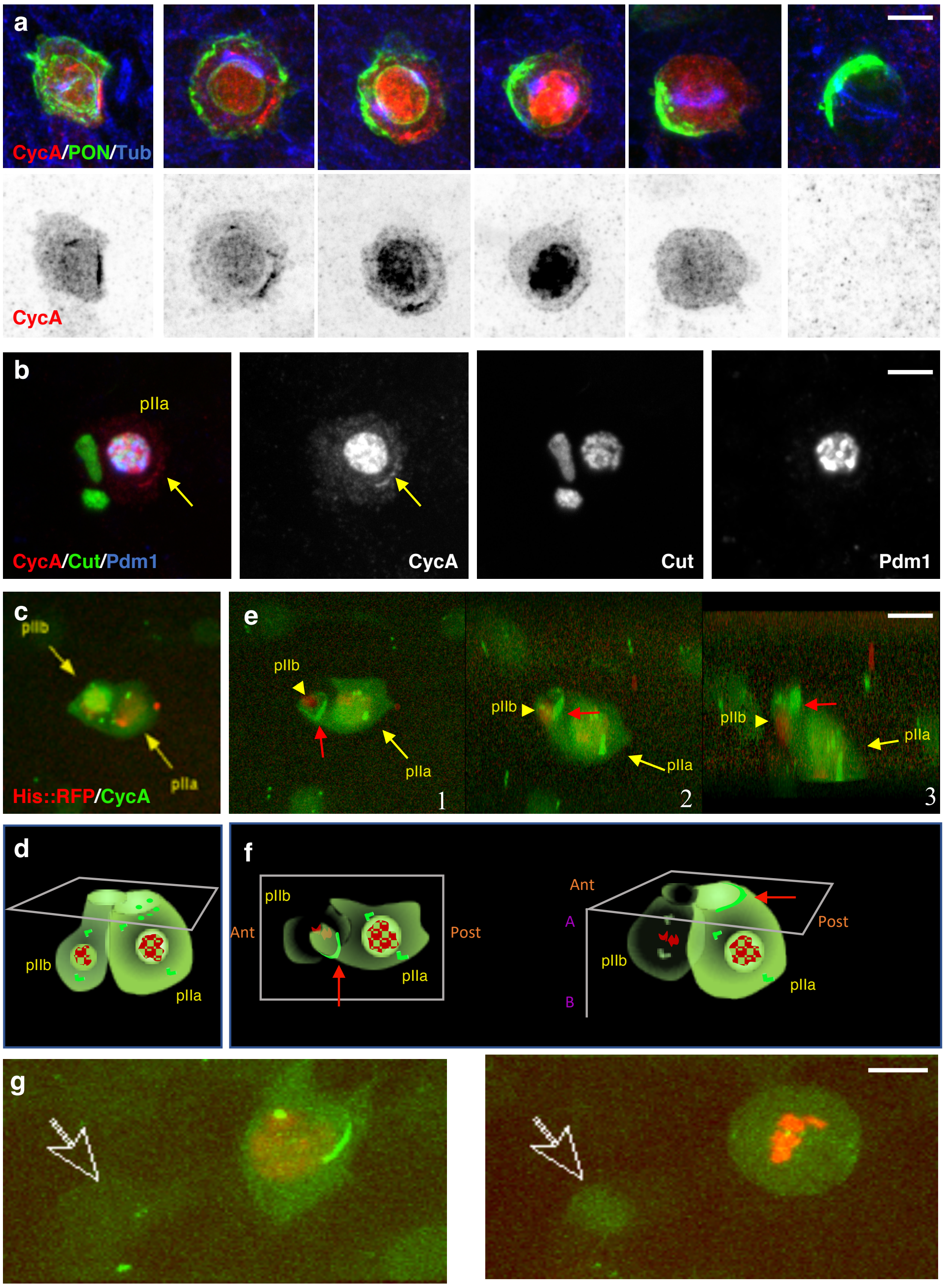

Supplementary figure 1: CycA localization in the bristle cell lineage and in epithelial cells. (a) Comparison of the PON::GFP and CycA crescent dynamics during pI mitosis in pupae 17 h APF. Top panel: PON::GFP (green), CycA (red) and tubulin (blue) immunostaining. Bottom panel: CycA alone in inverted color. Note that CycA crescent is formed before the PON::GFP crescent and does not last as long (n=5). (b) CycA crescent is present during pIIa cell division. Sensory cells are revealed by Cut immunoreactivity (green) and among them pIIa cell is identified by Pdm1 immunoreactivity (blue). Cyc(A) immunoreactivity is in red and the cortical posterior enrichment of CycA in pIIa prophase is pointed by a yellow arrow (n=3). (c-f) Snapshots of 4D live imaging of CycA::eGFP supplementary movie 2 during pIIb and pIIa mitosis. Representative cartoons are shown in d and f. CycA::eGFP is in green and H2B::RFP to reveal sensory cells in red. pIIb and pIIa are identified by their anterio-posterior position and size. (c) pIIb prophase and pIIa G2 phase. In pIIb, CycA is nuclear and cytoplasmic a landmark of prophase and no cortical CycA accumulation is detected. (d) schematic lateral view of c. (e) pIIb anaphase and pIIa prophase. 1- Dorsal view; 2 and 3 - 45° and 90° of lateral view. pIIb anaphase is shown by the condensed DNA and the absence of CycA is pointed by an arrowhead. CycA is detected in nucleus and cytoplasm of the pIIa cell (yellow arrow) and enrichment of cortical cycA (red arrow) is posterior and apical as shown on the panel 2 and 3. (f) Schematic representation of the dorsal view (Left panel) and of the lateral view (Right panel). Ant, anterior; Post, posterior; A, apical; B, basal. (g) Snapshots of 4D live imaging of CycA::eGFP supplementary movie 4 in epithelial cells. Epithelial cells are identified by the lack of H2B::YFP (red) and CycA is in green (white arrow). Note that no crescent is visualized in epithelial cell prophase identified by the nuclear CycA localization. Anterior on the left. Scale bars: 5 µm for a and g and 10 µm for b, c and e.

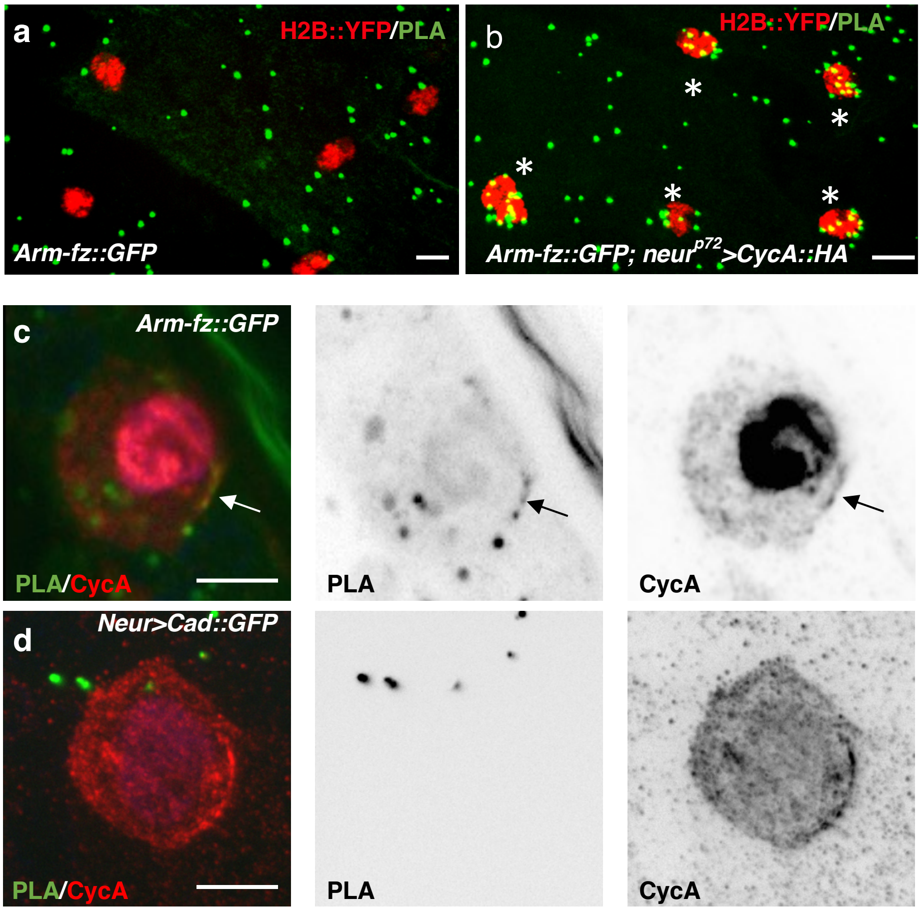

Supplementary figure 2: PLA analysis between CycA and Fz. CycA-GFP experiments to reveal epitope pairs (green, PLA dots) between CycA and Fz in a, b and c and between CycA and Cadherin in d. Many CycA-Fz epitope pairs (green dots indicated by asterisks) were observed on pI cells identified by the presence of H2B::YFP (red) after pI specific CycA overexpression in pupae 17 h APF expressing *arm-fz* (b), whereas much fewer dots are visible when CycA is not over-expressed (a). At a higher magnification, colocalization of CycA-Fz epitope pairs (green) and CycA immunoreactivity (red) are detected in mitotic pI cells of *arm-fz* pupae 17h APF (arrow in c). Coincident CycA-Cadherin epitope pairs (green dots) with CycA crescent (red) were never observed (d). Inverse fluorescence of separate color channels shown in right panels. n=13, 15, 4, and 4 in a, b, c, and d respectively. Posterior is to the right. Scale bars: 10 µm in a and b and 5 µm in c and d.

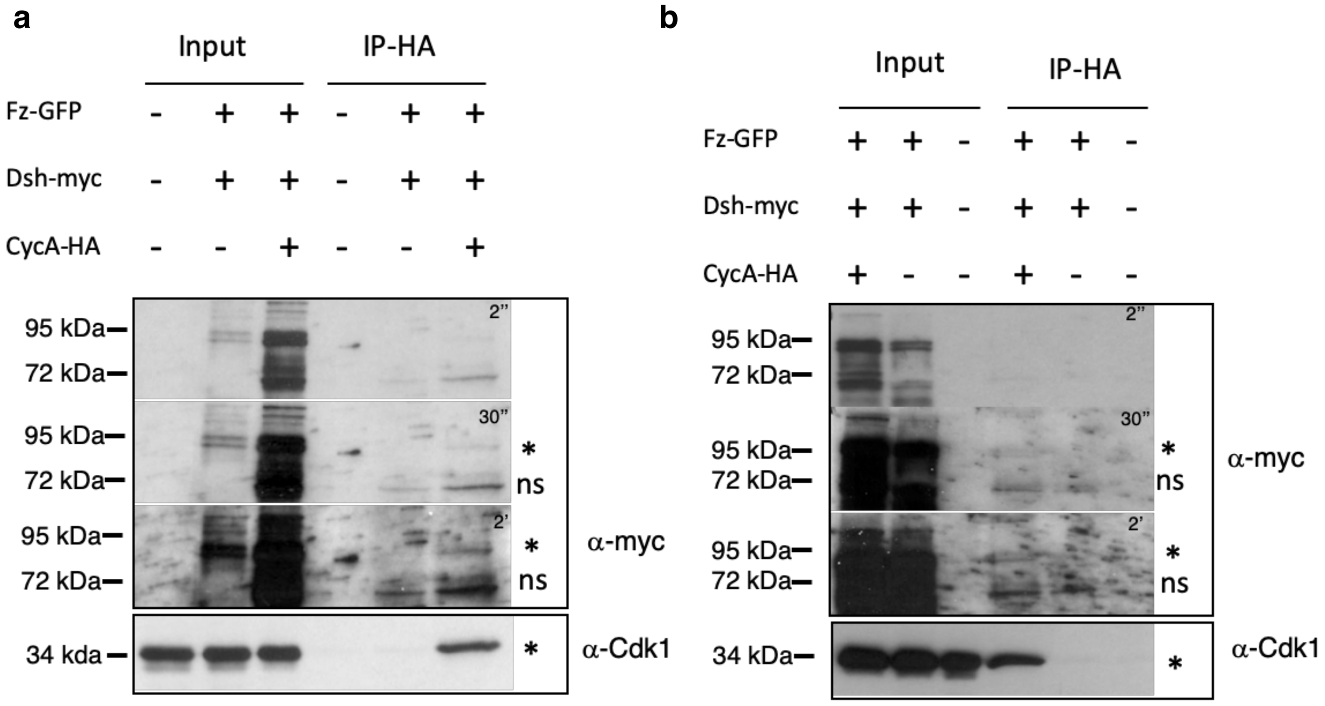

Supplementary figure 3: CycA co-precipitates with Dsh. Proteins immunoprecipitated using anti-HA beads were obtained from embryos expressing CycA::HA, Fz::GFP and Dsh::myc and as negative controls from embryos expressing only Fz::GFP and Dsh::myc or from w^{1118}. Dsh was revealed using anti-myc antibodies. Cdk1 detection was used as positive control. (a) Blot shown in Fig 2f. (b) Another experiment done in the same conditions as in (a). Three exposure times (2 sec, 30 sec and 2 min) are shown for the detection of Dsh::Myc proteins. Note that although Dsh::myc was less expressed in the input of embryos expressing only Fz::GFP and Dsh::myc, no bands were detected around 85 kDa in the corresponding co-immunoprecipitate even after 2 min of exposure whereas the non-specific (ns) bands were always detected.

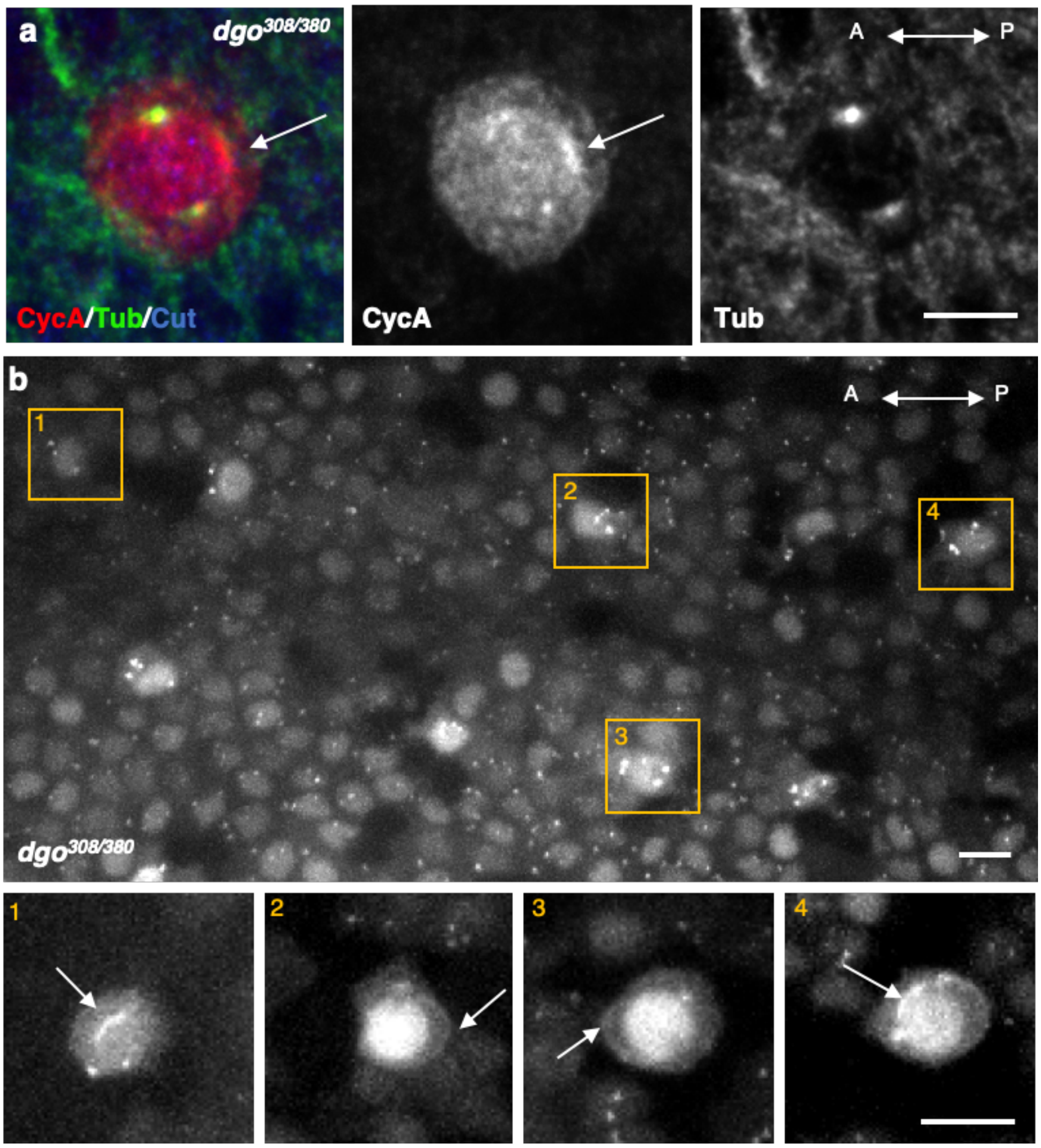

Supplementary figure 4: CycA cortical accumulation in *dgo* LOF. (a) Intracellular localization of endogenous CycA revealed by immunofluorescence (red) in $dgo^{308/380}$ (n=20). Sensory cells were identified using Cut immunostaining (blue) with γ-tubulin (green) also detected. Inverse fluorescence of separate color channels shown in right panels. Note the presence of a CycA apical-posterior crescent (b) CycA cortical accumulation was not restrained to the posterior part of pI cell. The top panel corresponds to the first slide of movie 7 and the bottom panel to snapshots of the pI cells (yellow squares in the top panel, as numbered) at the time of their division. Note that the CycA crescent is either anteriorly (pI cells 1, 3 and 4) or posteriorly (pI cell 2) located. Posterior is to the right. Scale bars, 10 µm.

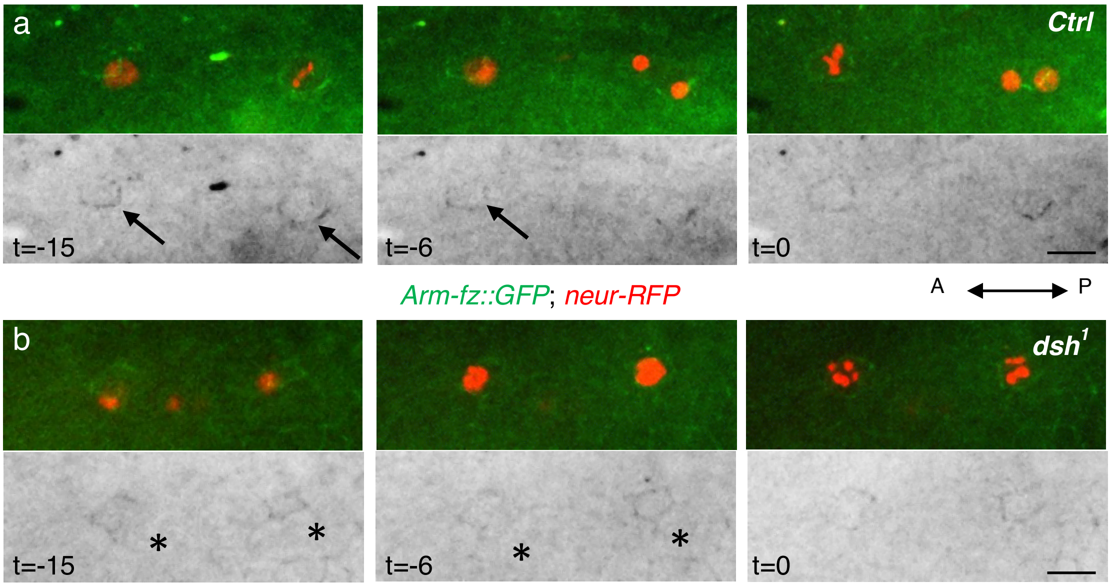

Supplementary figure 5: Dsh is required for the apical-posterior accumulation of Fz in pI cells. Time lapse-analysis of Fz::GFP (green in top panel and inverted color in bottom panel) in control (a) and *dsh*^{1} (b) pupae expressing H2B::RFP (red) under the control of *neur* to identify pI cells. Note that in the control Fz::GFP is posteriorly located (arrow) whereas in *dsh*^{1}, Fz is uniformly located at the apical cortex of pI cells (asterisk). Time before mitosis is given in minutes. Posterior on the right. Scale bars, 10 μm

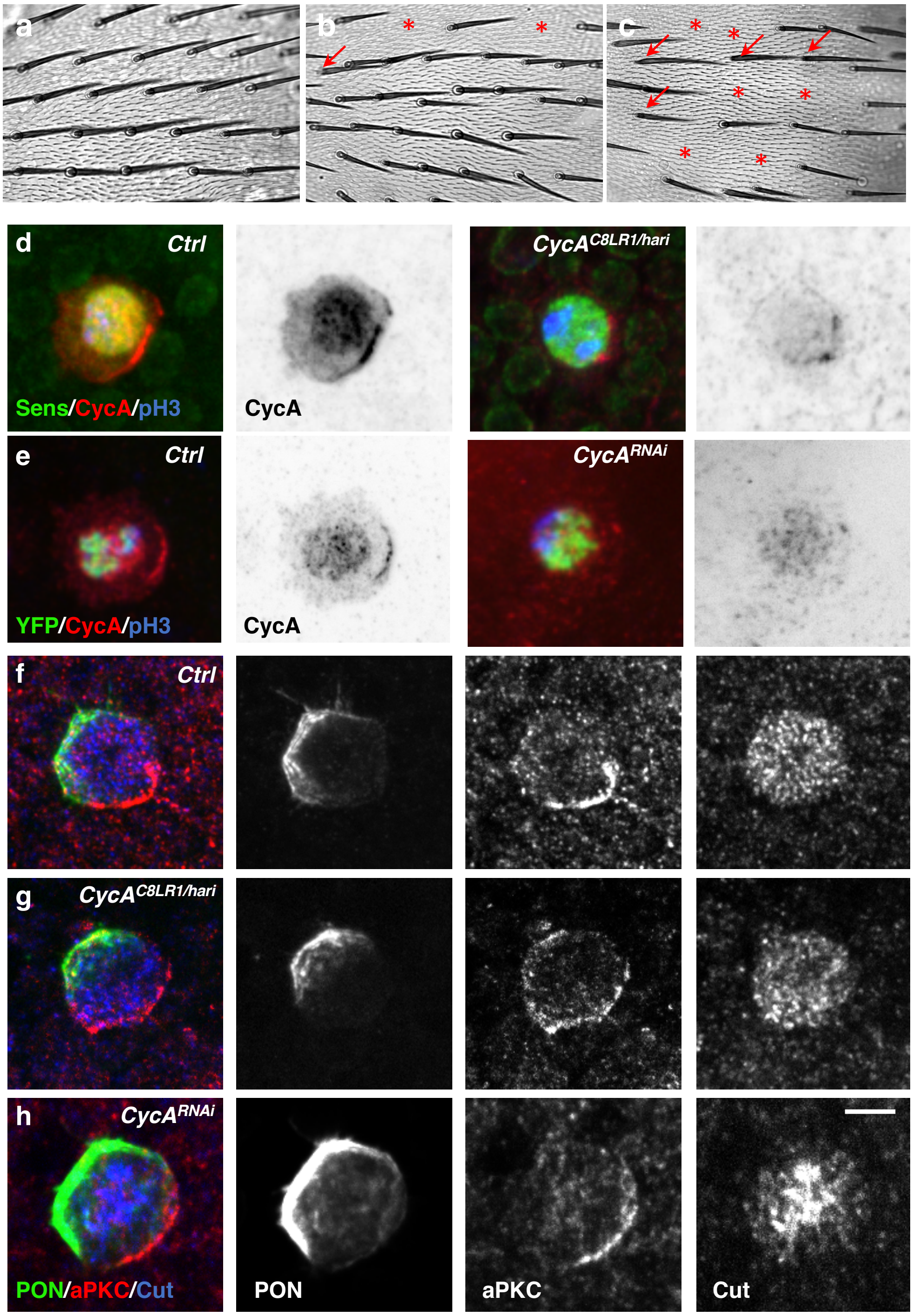

Supplementary figure 6: *CycA* LOF induced cell cycle arrest without affecting localization of cell fate determinants. (a-c) Micrograph of external view of sensory organs of control (a, n=10), $CycA^{C8LR1/hari}$ (b, n=12) and $CycA^{RNAi}$ (c, n=8) adult nota. Missing sensory organs are indicated by red stars and sensory organs without socket cells by red arrows. (d, e) CycA immunoreactivity (red and in grayscale) in prophase pI cells in $CycA^{C8LR1/hari}$ (d, n=5) and $CycA^{RNAi}$ (e, n=3). Sensory cells were identified using (b) Senseless (Sen) immunodetection and (c) *neur>H2B::YFP* (green). Mitotic cells were revealed using phospho-ser10 histone-3 (pH3) immunoreactivity (blue). In each context CycA immunoreactivity is compared with its control processed in the same experiment and imaged using the same settings. Note that in $CycA^{C8LR1/hari}$, a weak crescent is detected whereas in $CycA^{RNAi}$ no CycA apical accumulation was detected. (f, h) Asymmetry of the pI cell is not impaired in $CycA^{C8LR1/hari}$ (g, n=3) and $CycA^{RNAi}$ (h, n=4) compared to control (f) in pupae 17 h APF. PON::GFP is in green, and the posterior fate determinant aPKC in red. Note that in $CycA^{C8LR1/hari}$ the asymmetric localizations were similar to those observed in the control. Posterior on the right. Scale bars, 5 µm.

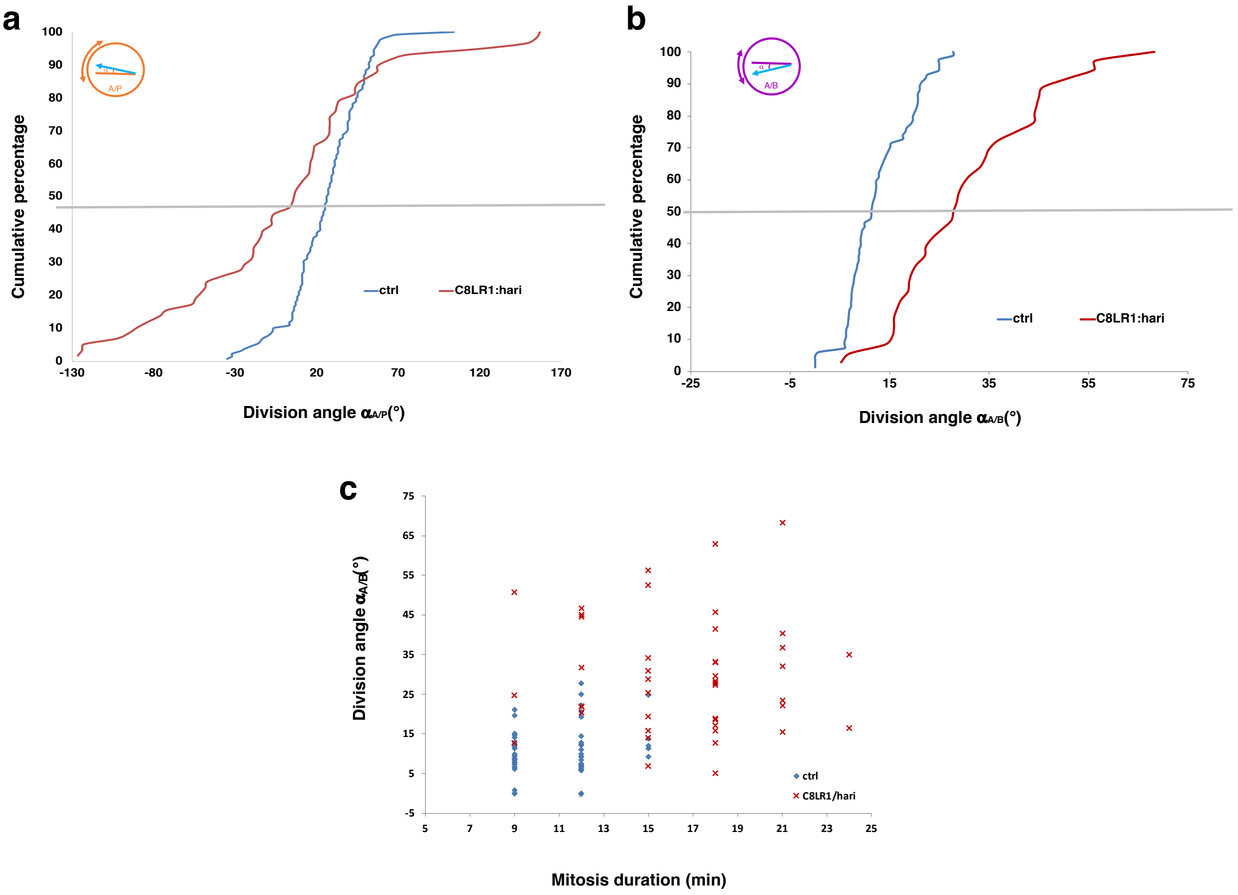

Supplementary figure 7: *CycA* LOF induced mis-orientation of pIIa cell division. Angles of the mitotic spindle relative to both the antero-posterior axis ($\alpha_{A/P}$, a) and to the apico-basal axis ($\alpha_{A/B}$, b) in $CycA^{C8LR1/hari}$. Cumulative plots of $\alpha_{A/P}$ (a) and of $\alpha_{A/B}$ (b) in control (n=128 in (a) and n=57 in (b)) and in $CycA^{C8LR1/hari}$ (n=84 in (a) and n=36 in (b)). The horizontal axis represents the angle between the axis of division and the midline in (a) or the epithelial plane in (b) and the vertical axis the cumulative percentage of cells. Along the antero-posterior axis, measured angles are positive when the anterior spindle pole is closer to the midline than the posterior spindle pole. Along the apico-basal axis, measured angles are positive when the anterior spindle pole is more basal than the posterior spindle pole. Significance was determined by an unpaired two-tailed Mann-Whitney-test for $\alpha_{A/P}$ angles and by a Wilcoxon-test for $\alpha_{A/B}$ angles. (c) Relationship between mitosis duration and the $\alpha_{A/B}$ angles in control (n=55) and in $CycA^{C8LR1/hari}$ (n=44) pIIa cells. Note that, although the duration of mitoses in $CycA^{C8LR1/hari}$ was longer there is no correlation between mitosis duration and orientation of the division. Source data are provided as a Source Data file.

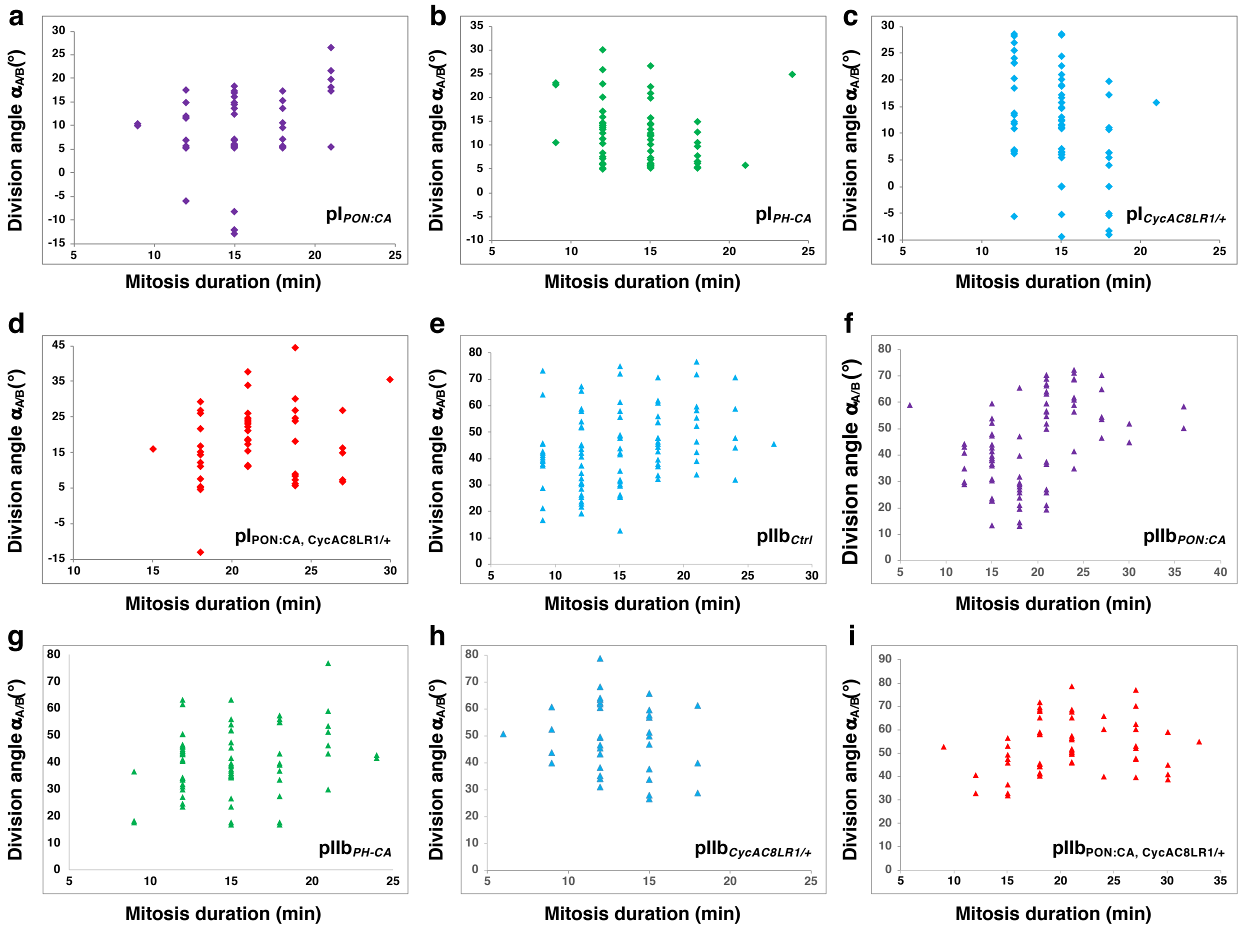

Supplementary figure 8. Spindle orientation during pI and pIIb mitosis is independent of the mitosis duration. (a-d) Relationship between mitosis duration and the $\alpha_{A/B}$ angles of pI cells in *PON-CA* (a, n=48), *PH-CA* (b, n=71), *CycA*$^{C8IR1/+}$ (c, n=77) and *PON-CA;CycA*$^{C8IR1/+}$ (d, n=48). (e-i) Relationship between mitosis duration and the $\alpha_{A/B}$ angles in pIIb cells in control (e, n=97), *PON-CA* (f, n=81), *PH-CA* (g, n=70), *CycA*$^{C8IR1/+}$ (h, n=37) and *PON-CA;CycA*$^{C8IR1/+}$ (i, n=60). Note that for each condition, no correlation is observed between the mitosis duration and the angle measured. Source data are provided as a Source Data file.

Supplementary table 1: Sequence of oligonucleotides used in this study.

	**Primers used to amplify the 5' and 3' fragments of the CycA gene**
Forward-5'CycA	5'GTCTACGGAGCGACAATTCAATTC3'
Reverse-5'CycA-PON	5'CGCGCGGGTGCCTTCAGCGTAATCTGGAAC3'
Reverse-5'CycA-PH	5'GATGTTCCAGATTACGCTGCAGGATGATGAGG3'
Forward-3'PON-CA	5'GCAACCGCCAAGTCCCCCATGGCCAGTTTCC3'
Forward-3'PH-CA	5'CCGGTCGCCACCTCCCCCATGGCCAGTTTCC3'
Reverse-3'CycA	5'CTGAACCACTTCCAGGAACC3'
	**Overlapping primers used to amplify the PON and PH fragments**
Forward CycA-PON	5'GTTCCAGATTACGCTGAAGGCACCCGCGCG3'
Reverse PON-CycA	5'GGAAACTGGCCATGGGGGACTTGGCGGTTGC3'
Forward CycA-PH	5'GATGTTCCAGATTACGCTGCAGGATGATGAGG3'
Reverse PH-CycA	5'GGAAACTGGCCATGGGGGAGGTGGCGACCGG3'

Supplementary Table 2: Fly genotypes. Fly genotypes used in each figure and movie.

**Figure 1**	A-C	*neur>UAS-H2B ::YFP*
	D, E	*pneurD-H2B::RFP ; CycA::eGFP*
**Figure 2**	A-J	*arm-fz::GF*P
	K, L	w^{1118}<br>*Arm-fz ::GFP/dsh-dsh::Myc ; Da-Gal4/UAS-CycA::HA*<br>*Arm-fz ::GFP/dsh-dsh::Myc*
**Figure 3**	A	w^{1118}
	B	fz^{K21}/fz^{KD4}
	C	dsh^{1}/Y
	D	*pneurD-H2B ::RFP ; CycA::eGFP / +*
	E	dsh^{1}/Y*; pneurD-H2B ::RFP ; CycA::eGFP / +*
	F-G	*neur>UAS-fz ::myc*
**Figure 4**	B, E, G, H, I	*neur>UAS-H2B ::RFP, UAS-PON ::GFP/+*
	C, F, G, H, I	*pneuD-H2B ::RFP/+ ;neur>UAS-PON ::GFP,* $CycA^{C8LR1}/CycA^{hari}$
	G, H, J	*tubulin-*$Gal80^{ts}$ */+ ; neur> UAS-his::RFP, UAS-pon::GFP/+*
	G, H, J	*UAS-*$CycA^{RNAi}$*, tub-*$Gal80^{ts}$*/+ ; neur> UAS-his::RFP, UAS-pon::GFP/+*
**Figure 5**	A, D-H, K, L	*neur>UAS-H2B ::RFP, UAS-PON ::GFP /+*
	B, D-G, I, K, L	*UAS-PON-CA / + ; neur>UAS-H2B ::RFP, UAS-PON ::GFP /+*
	C, D-G, J, K	*UAS-PHCA / neur>UAS-H2B ::RFP, UAS-PON ::GFP*

	F, G, L	*CycA*^{C8LR1} */ neur>UAS-H2B ::RFP, UAS-PON ::GFP*
	F, G, L	*UAS-PON-CA / + ; CycA*^{C8LR1} */ neur>UAS-H2B ::RFP, UAS-PON ::GFP*
**Figure 6**	A, C	*mud::GFP[50E1]/+ ; mud::GFP[62E1], mud::GFP[65B2] / neur<UAS-H2B ::RFP*
	B, C	*mud::GFP[50E1]/ UAS-CycA*^{RNAi}*, tub-Gal80ts ; mud::GFP[62E1], mud::GFP[65B2] / neur>UAS-H2B::RFP*
	D, G, H, I	*neur>UAS-PON ::GFP*
	E, G, J, K	*neur>UAS-PON ::GFP, CycA*^{C8LR1}*/CycA*^{hari}
	F, G	*dsh*^{1}*/Y ; neur>UAS-PON ::GFP*
**Supplementary figure 1**	A	*neur>UAS-PON::GFP/+*
	B	*w*^{1118}
	C, E, G	*pneurD-H2B::RFP ; CycA::eGFP/ +*
**Supplementary figure 2**	A, C	*arm-fz::GFP*
	B	*arm-fz::GFP;neur>UAS-CycA::HA*
	D	*DE-Cadherin::GFP*
**Supplementary figure 3**	A, B	*w*^{1118}<br>*Arm-fz ::GFP/dsh-dsh::Myc ; Da-Gal4/UAS-CycA::HA*<br>*Arm-fz ::GFP/dsh-dsh::Myc*
**Supplementary figure 4**	A	*dgo*^{308}*/dgo*^{380}
	B	*dgo*^{308} *; CycA::eGFP /dgo*^{380} *; CycA::eGFP*
**Supplementary figure 5**	A	*Arm-Fz::GFP*
	B	*dsh*^{1}*; Arm-Fz::GFP*
**Supplementary figure 6**	A	*w*^{1118}
	B	*CycA*^{C8LR1}*/CycA*^{hari}
	C	*neur> UAS-CycA*^{RNAi}
	D	*neur>UAS-PON ::GFP*
	E	*neur>UAS-PON ::GFP, CycA*^{C8LR1}*/CycA*^{hari}
	F	*UAS-CycARNAi, tub-Gal80*^{ts}*/+ ; neur> UAS-PON::GFP/+*
**Supplementary figure 7**	A,B,C	*tubulin-Gal80*^{ts} */+ ; neur> UAS-his::RFP, UAS-pon::GFP/+*
	A,B,C	*pneuD-H2B ::RFP/+ ;neur>UAS-PON ::GFP, CycA*^{C8LR1}*/CycA*^{hari}
**Supplementary figure 8**	A, F	*UAS-PON-CA / + ; neur>UAS-H2B ::RFP, UAS-PON ::GFP /+*
	B, G	*UAS-PHCA / neur>UAS-H2B ::RFP, UAS-PON ::GFP*
	C, H	*CycA*^{C8LR1} */ neur>UAS-H2B ::RFP, UAS-PON ::GFP*
	D, I	*UAS-PON-CA / + ; CycA*^{C8LR1} */ neur>UAS-H2B ::RFP, UAS-PON ::GFP*
	E	*neur>UAS-H2B ::RFP, UAS-PON ::GFP /+*
**Movie 1**		*pneurD-H2B::RFP ; CycA::eGFP*
**Movie 2**		*pneurD-H2B::RFP ; CycA::eGFP*
**Movie 3**		*pneurD-H2B::RFP ; CycA::eGFP*
**Movie 4**		*pneurD-H2B::RFP ; CycA::eGFP*

**Movie 5**		*dgo^{308} ; CycA::eGFP/ dgo^{380} ; CycA::eGFP*
**Movie 6**		*dgo^{308} ; CycA::eGFP/ dgo^{380} ; CycA::eGFP*
**Movie 7**	left	*pneurD-H2B::RFP ; CycA::eGFP*
	right	*dsh^{1}/Y; pneurD-H2B ::RFP ; CycA::eGFP / +*
**Movie 8**		*Arm-Fz::GFP*
**Movie 9**		*Dsh^{1}; Arm-Fz::GFP*
**Movie 10**	left	*mud::GFP[50E1]/+ ; mud::GFP[62E1], mud::GFP[65B2] / neur<UAS-H2B ::RFP*
	right	*mud::GFP[50E1]/ UAS-CycA^{RNAi}, tub-Gal80ts ; mud::GFP[62E1], mud::GFP[65B2] / neur>UAS-H2B::RFP*

Uncropped scan of blot corresponding to the immuno-precipitation experiment shown in supplementary figure 3b.

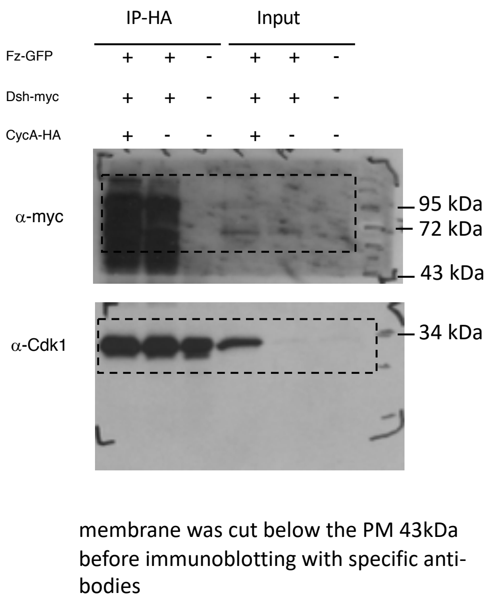

Same legend of supplementary figure 3b
