# Supplementary material for: Cortical Cyclin A controls spindle orientation during asymmetric cell divisions in Drosophila
Source: Nat Commun. 2022 May 17;13:2723. doi: 10.1038/s41467-022-30182-1 (PMC9114397; doi:10.1038/s41467-022-30182-1)
Supplement: Supplementary file 3 — Description of Additional Supplementary Files [file 41467_2022_30182_MOESM3_ESM.pdf]

Description of Additional Supplementary Files

File name: Supplementary Movie 1

Description: In *vivo* imaging of a control sensory cell to follow the intracellular localization of CycA::eGFP during pI division at 16-17 h APF. Sensory cells are identified by the expression of H2B-RFP (blue). The movie was segmented to separate apical and basal CycA into green and red signals respectively. Posterior is to the right and the view is dorsal. Each frame was obtained by combining a z-stack (composed of optical sections separated by 1µm) acquired every 2 minutes. Posterior on the right.

File name: Supplementary Movie 2

Description: In *vivo* imaging of control sensory cells to follow the intracellular localization of CycA::eGFP during pIIb and pIIa division at 18-20h APF. A CycA crescent is formed during the pIIa division, but is not observed during pIIb division. Posterior is to the right and the view is dorsal. Each frame was obtained by combining a z-stack (composed of optical sections separated by 1µm) acquired every 3 minutes. Posterior on the right.

File name: Supplementary Movie 3

Description: In *vivo* imaging of control sensory cell pIIIb division to follow the intracellular localization of CycA::eGFP starting at 16 h APF. CycA crescent is formed during the pI and pIIa division, but is never observed during pIIb and pIIIb division. Posterior is to the right and the view is dorsal. Each frame was obtained by combining a z-stack (composed of optical sections separated by 1µm) acquire every 3 minutes. Posterior on the right.

File name: Supplementary Movie 4

Description: In *vivo* imaging of a pI cell and surrounding epithelial cell to follow the intracellular localization of CycA::eGFP at 16-17 h APF. H2B::YFP appears red and CycA green (white arrow). Epithelial cells are identified by the lack of H2B::YFP. Note that no cortical apical enrichment of CycA is observed during epithelial cell division. Posterior on the right.

File name: Supplementary Movie 5

Description: In *vivo* imaging of a pI cell to follow the intracellular localization of CycA::eGFP in the *dgo*$^{308/380}$ mutant at 16-17 h APF. Note that CycA crescent is visible in *dgo*$^{308/380}$ mutant pI cell. Posterior on the right.

File name: Supplementary Movie 6

Description: In *vivo* imaging to follow the intracellular localization of CycA::eGFP in the *dgo*$^{308/380}$ mutant pupae at 16-17 h APF. Note that the CycA crescent (arrow) is misoriented related to A/P axis in some pI cells. Posterior on the right.

File name: Supplementary Movie 7

Description: In *vivo* imaging of a pI cell to follow the intracellular localization of CycA::eGFP in the heterozygous control (left) and *dsh*^{1} (right) mutant at 16-17 h APF. Sensory cells are identified by the expression of H2B-RFP (red). Note that no CycA crescent is visible in *dsh*^{1} mutant pI cells. Posterior on the right.

File name: Supplementary Movie 8

Description: In *vivo* imaging of a pI cells at 16-17 h APF to follow the intracellular localization of Fz::GFP. Right: Fz (green) and sensory cells identified by the expression of H2B-RFP (red). Left: Fz channel alone. Note that before mitosis posterior accumulation of Fz::GFP is visible in pI cells. Posterior on the right.

File name: Supplementary Movie 9

Description: In *vivo* imaging of a pI cells at 16-17 h APF to follow the intracellular localization of Fz::GFP in *dsh*^{1} mutant pupae. Right: Fz (green) and sensory cells identified by the expression of H2B-RFP (red). Left: Fz channel alone. Note that no posterior accumulation of Fz::GFP is visible in *dsh*^{1} mutant pI cells. Posterior on the right.

File name: Supplementary Movie 10

Description: In *vivo* imaging of a pI cell at 16-17 h APF to follow the intracellular localization of Mud::eGFP in the control (left) and *CycA*^{RNAi} (right) mutant. Sensory cells are identified by the expression of H2B-RFP (red). Note that no posterior Mud crescent is visible in *CycA*^{RNAi} mutant pI cells. Posterior on the right.
