# Supplementary material for: Cortical Cyclin A controls spindle orientation during asymmetric cell divisions in Drosophila
Source: Nat Commun. 2022 May 17;13:2723. doi: 10.1038/s41467-022-30182-1 (PMC9114397; doi:10.1038/s41467-022-30182-1)
Supplement: Supplementary file 14 — Reporting Summary [file 41467_2022_30182_MOESM14_ESM.pdf]

# nature portfolio

Corresponding author(s): AUDIBERT AGNES

Last updated by author(s): Nov 3, 2022

# Reporting Summary

Nature Portfolio wishes to improve the reproducibility of the work that we publish. This form provides structure for consistency and transparency in reporting. For further information on Nature Portfolio policies, see our Editorial Policies and the Editorial Policy Checklist.

## Statistics

For all statistical analyses, confirm that the following items are present in the figure legend, table legend, main text, or Methods section.

| n/a | Confirmed | |
|---|---|---|
| ☐ | ☒ | The exact sample size (*n*) for each experimental group/condition, given as a discrete number and unit of measurement |
| ☐ | ☒ | A statement on whether measurements were taken from distinct samples or whether the same sample was measured repeatedly |
| ☐ | ☒ | The statistical test(s) used AND whether they are one- or two-sided<br>*Only common tests should be described solely by name; describe more complex techniques in the Methods section.* |
| ☒ | ☐ | A description of all covariates tested |
| ☒ | ☐ | A description of any assumptions or corrections, such as tests of normality and adjustment for multiple comparisons |
| ☐ | ☒ | A full description of the statistical parameters including central tendency (e.g. means) or other basic estimates (e.g. regression coefficient) AND variation (e.g. standard deviation) or associated estimates of uncertainty (e.g. confidence intervals) |
| ☐ | ☒ | For null hypothesis testing, the test statistic (e.g. $F$, $t$, $r$) with confidence intervals, effect sizes, degrees of freedom and $P$ value noted<br>*Give P values as exact values whenever suitable.* |
| ☒ | ☐ | For Bayesian analysis, information on the choice of priors and Markov chain Monte Carlo settings |
| ☒ | ☐ | For hierarchical and complex designs, identification of the appropriate level for tests and full reporting of outcomes |
| ☒ | ☐ | Estimates of effect sizes (e.g. Cohen's $d$, Pearson's $r$), indicating how they were calculated |

*Our web collection on statistics for biologists contains articles on many of the points above.*

## Software and code

Policy information about availability of computer code

| | |
|---|---|
| Data collection | Metamorph Serie 7.10.3.279 |
| Data analysis | Adobe photoshop CS6 extended (Version 13 x64)<br>Huygens Essentiel (version 2017, STED option).<br>Image J2 software (version 1.53a)<br>Fiji software (ImageJ2, version 2.3.0/153f)<br>Excel pack office (version 16.16.27)<br>Kaleidagraph (version 4.5.4)<br>Adobe photoshop CS6 extended (Version 13 x64)<br>Image J2 software (version 1.53a)<br>Fiji software (ImageJ2, version 2.3.0/153f)<br>Excel pack office (version 16.16.27)<br>Kaleidagraph (version 4.5.4) |

For manuscripts utilizing custom algorithms or software that are central to the research but not yet described in published literature, software must be made available to editors and reviewers. We strongly encourage code deposition in a community repository (e.g. GitHub). See the Nature Portfolio guidelines for submitting code & software for further information.

## Data

Policy information about availability of data

All manuscripts must include a data availability statement. This statement should provide the following information, where applicable:

- Accession codes, unique identifiers, or web links for publicly available datasets
- A description of any restrictions on data availability
- For clinical datasets or third party data, please ensure that the statement adheres to our policy

A list of figures that have asociated raw data

# Field-specific reporting

Please select the one below that is the best fit for your research. If you are not sure, read the appropriate sections before making your selection.

☒ Life sciences ☐ Behavioural & social sciences ☐ Ecological, evolutionary & environmental sciences

For a reference copy of the document with all sections, see nature.com/documents/nr-reporting-summary-flat.pdf

# Life sciences study design

All studies must disclose on these points even when the disclosure is negative.

| | |
|---|---|
| Sample size | For quantitative analysis, the number of cells varies between 80 to 128. No statistical method was used to predetermined the sample size. The sample size was based on that to use by all other similar studies in the field. Information about the simple size are mentioned in main text, figures legends and source data. |
| Data exclusions | Data were not excluded from the analysis |
| Replication | All sample were obtained from at least three independent animals and in all cases successfully<br>For qualitative data, representative experiments are illustrated in the manuscript |
| Randomization | Experiment were randomized. pI cells were analyzed on at least three independent Pupae. Crosses were to obtain pupae were repeated. All the quantifications were done independently at least by two authors. |
| Blinding | The result of each experiment was collected and analyzed by the person in charge of the experiment. In experiments, the genotype determination required fly chromosomes markers recognition, thus blinding was not possible but samples were selected randomly and unbiased manner. |

# Reporting for specific materials, systems and methods

We require information from authors about some types of materials, experimental systems and methods used in many studies. Here, indicate whether each material, system or method listed is relevant to your study. If you are not sure if a list item applies to your research, read the appropriate section before selecting a response.

## Materials & experimental systems

| n/a | Involved in the study | |
|---|---|---|
| ☐ | ☒ | Antibodies |
| ☒ | ☐ | Eukaryotic cell lines |
| ☒ | ☐ | Palaeontology and archaeology |
| ☐ | ☒ | Animals and other organisms |
| ☒ | ☐ | Human research participants |
| ☒ | ☐ | Clinical data |
| ☒ | ☐ | Dual use research of concern |

## Methods

| n/a | Involved in the study | |
|---|---|---|
| ☒ | ☐ | ChIP-seq |
| ☒ | ☐ | Flow cytometry |
| ☒ | ☐ | MRI-based neuroimaging |

## Antibodies

| | |
|---|---|
| Antibodies used | rabbit anti-aPKCzeta (Tebu- Santa-Cruz, sc-216, 1:500), mouse anti-Cut (DSHB, #2B10, 1:500); rabbit anti-GFP (Santa-Cruz Biotechnology, #sc- 8334; 1:500); mouse anti-GFP (Roche, No 11 814 460 001, 1:500); rabbit anti-GFP (Abcam, Ab290, 1/500), rabbit anti-CycA (a gift from P. O'Farrell (UCSF, CA, USA), 1:500); rat anti-Dsh (gift from T. Uemura, 1/500), rabbit anti-Mud (gift from Y. Bellaïche, 1:500), mouse anti-Myc (Roche, clone 9E10, 1:500), rabbit anti-Myc (Merck, 06-549, 1:1000), rabbit anti-Pdm1 (gift from T. Préat; École Supérieure de Physique et de Chimie Industrielles, Paris, France; 1:200), rat anti-pTyr (R&D system, MAB 16761, 1:500), and rat anti-γ–tubulin (gift from M.H. Verlhac, 1:500), rat-anti-Sens (1:1000, gift of Y. Bellaïche, Institute Curie, Paris, France), |

rabbit anti-phospho-Histone H3 (Upstate, 06-570, 1:10000), anti-HA (rat, Roche, 11867423001, 1/500), anti-Cdk1 (PSTAIR, rabbit, 06-923, Merck-Millipore, 1/2000),
Alexa 488-conjugated secondary anti-mouse (#A11029), anti-rat (#A11006), anti-rabbit (#A11034), Alexa 568-conjugated secondary anti-mouse (#A11031), anti-rat (#A11077), and anti-rabbit (#A11011) from Molecular Probes and used at 1:1000. Sigma Aldrich, Anti-Rabbit Abberior? Star 635 (Sigma Aldricht, 1/100). Cy5-conjugated antibodies anti-mouse (#715-175-151), anti-rat (#712-175-153), or anti-rabbit (#711-175-152) were purchased from Jackson Immunoresearch and were used at 1:2000. HRP conjugated antibodies anti rat (Abcam, Ab6734, 1/5000) and HRP conjugated antibodies anti rabbit (Jackson Immunoresearch, 711-035-152, 1/10000).

Validation

The validation of antibodies can be found either on the manufacturer's website or in the listed articles:

Anti -Cut: https://dshb.biology.uiowa.edu/2B10
Anti GFP rabbit: https://www.abcam.com/gfp-antibody-ab290.html
Anti GFP rabbit: https://www.scbt.com/p/gfp-antibody-fl?requestFrom=search
Anti GFP mouse:
https://www.sigmaaldrich.com/FR/fr/product/roche/11814460001?gclid=Cj0KCQiA95aRBhCsARIsAC2xvfw-0_o2a7Lvn_40j4KjEnSBdMt5I6hTg2exel06CDDCDAhKSVceT-UaAsbwEALw_wcB
Anti Myc mouse: https://www.sigmaaldrich.com/FR/fr/product/roche/roamyc
Anti Myc rabbit: https://www.merckmillipore.com/FR/fr/product/Anti-Myc-Tag-Antibody,MM_NF-06-549#documentation
Anti pTyr rat: https://www.rndsystems.com/products/phospho-tyrosine-antibody-216954_mab16761
anti-phospho-Histone H3 rabbit:
https://www.sigmaaldrich.com/FR/fr/product/mm/06570?gclid=Cj0KCQiA95aRBhCsARIsAC2xvfyjjAicQyXvBBcc0p2GX3sCNqQbN5j_nNHYPKINsMJlCXIy3rXTNvQaAivcEALw_wcB
Anti HA rat:
https://www.sigmaaldrich.com/FR/fr/product/roche/roahaha?gclid=CjwKCAiA1JGRBhBSEiwAxXblwZMaRle_LwSZ98Nhig1CMW2_ps8vNcyVduQQP6kZNPg2FGkMgHsffRoCRh4QAvD_BwE
Anti-CdK1 rabbit: https://www.merckmillipore.com/FR/fr/product/Anti-Cdk1-Cdc2-PSTAIR-Antibody,MM_NF-06-923?ReferrerURL=https%3A%2F%2Fwww.google.com%2F

Anti aPKCzeta: Anti-aPKCzeta Tebu- Santa-Cruz catalog#: sc-216
https://www.scbt.com/fr/p/pkc-zeta-antibody-c-20
Yohanns Bellaïche, Anna Radovic, Daniel F. Woods, Cahir J. O'Kane, Peter J. Bryant, François Schweisguth. The Partner of Inscuteable/Discs-Large Complex Is Required to Establish Planar Polarity during Asymmetric Cell Division in Drosophila Cell, Volume 106, ISSUE 3, P355-366, , 2001 - DOI:https://doi.org/10.1016/S0092-8674(01)00444-5

Anti-CycA:
Kai Yuan, Jeffrey A. Farrell, Patrick H. O'Farrell - Different cyclin types collaborate to reverse the S-phase checkpoint and permit prompt mitosis J Cell Biol (2012) 198 (6): 973–980.
https://doi.org/10.1083/jcb.201205007

Lehner C.F., O'Farrell P.H. Expression and function of Drosophila cyclin A during embryonic cell cycle progression. Cell. 56:957–968 1989 - https://doi.org/10.1016/0092-8674(89)90629-6

Sallé J, Campbell SD, Gho M, Audibert A. CycA is involved in the control of endoreplication dynamics in the Drosophila bristle lineage. Development. 2012 Feb;139(3):547-57. doi: 10.1242/dev.069823.

Anti Dsh
Shimada Y, Usui T, Yanagawa S, Takeichi M, Uemura T. Asymmetric colocalization of Flamingo, a seven-pass transmembrane cadherin, and Dishevelled in planar cell polarization. Curr Biol. 2001 Jun 5;11(11):859-63. doi: 10.1016/s0960-9822(01)00233-0.

Anti Mud
Ségalen M, Johnston CA, Martin CA, Dumortier JG, Prehoda KE, David NB, Doe CQ, Bellaïche Y. The Fz-Dsh planar cell polarity pathway induces oriented cell division via Mud/NuMA in Drosophila and zebrafish. Dev Cell. 2010 Nov 16;19(5):740-52. doi: 10.1016/j.devcel.2010.10.004.

Anti p-Tyr
Roland Le Borgne, Yohanns Bellaïche, François Schweisguth. Drosophila E-cadherin regulates the orientation of asymmetric cell division in the sensory organ lineage. Curr Biol. 2002 Jan 22;12(2):95-104. doi: 10.1016/s0960-9822(01)00648-0.

Anti-pdm1
Simon F, Fichelson P, Gho M, Audibert A. Notch and Prospero repress proliferation following cyclin E overexpression in the Drosophila bristle lineage. PLoS Genet. 2009 Aug;5(8):e1000594. doi: 10.1371/journal.pgen.1000594. Epub 2009 Aug 7.

Anti rat anti-γtubulin
Brunet S, Polanski Z, Verlhac MH, Kubiak JZ, Maro B. Bipolar meiotic spindle formation without chromatin. Curr Biol. 1998 Nov 5;8(22):1231-4. doi: 10.1016/s0960-9822(07)00516-7.

Anti Sens
Antibodies was made in Y. Bellaïche lab. Senseless expression profile obtained with these antibodies was similar to that observed with antibodies anti-sens made in Guinea-pig in H. Bellen lab.

Nolo R, Abbott LA, Bellen HJ. Senseless, a Zn finger transcription factor, is necessary and sufficient for sensory organ development in

Drosophila. Cell. 2000 Aug 4;102(3):349-62. doi: 10.1016/s0092-8674(00)00040-4.
Ayeni JO, Audibert A, Fichelson P, Srayko M, Gho M, Campbell SD. G2 phase arrest prevents bristle progenitor self-renewal and synchronizes cell division with cell fate differentiation. Development. 2016-143:1160-9.

## Animals and other organisms

Policy information about studies involving animals; ARRIVE guidelines recommended for reporting animal research

| | |
|---|---|
| Laboratory animals | Drosophila melanogaster - males and females – embryos (0 to 24h after hatching) - pupal stages (between 4 to 6 days after hatching) and adults (2 to 4 days old) |
| Wild animals | The study does not involve wild animals |
| Field-collected samples | Samples were not collected from the field |
| Ethics oversight | The study requires the use of laboratory Drosophila melanogaster flies and we have respected all ethical rules concerning this organism. |

Note that full information on the approval of the study protocol must also be provided in the manuscript.
